# Preparation and Evaluation of a Novel Branched Polymer as Thickener for Calcium Chloride-Based Drilling and Completion Fluids

**DOI:** 10.3390/molecules29235542

**Published:** 2024-11-23

**Authors:** Xianbin Zhang, Zhongfeng Yang, Qian Wang, Weijie Chen, Tengjiao Liu, Tao Zhou, Shulin Li, Tongle Xin, Jie Cao, Xia Xin

**Affiliations:** 1 Tianjin Key Laboratory of Complicated Conditions Drilling Fluids, Tianjin 300280, China; zhangxbin@cnpc.com.cn (X.Z.); yangzfeng@cnpc.com.cn (Z.Y.); bh-wangqian@cnpc.com.cn (Q.W.); sharonvv@126.com (W.C.); liutengjiao8@cnpc.com.cn (T.L.); zhou_tao@cnpc.com.cn (T.Z.); 2 Key Laboratory of Unconventional Oil & Gas Development (China University of Petroleum (East China)), Ministry of Education, Qingdao 266580, China; z23020092@s.upc.edu.cn; 3 National Engineering Research Center for Colloidal Materials, School of Chemistry and Chemical Engineering, Shandong University, Jinan 250100, China; 202320352@mail.sdu.edu.cn

**Keywords:** drilling fluid, rheological performance, thickener, branched polymer

## Abstract

Calcium halide-based fluids are often used in drilling and completion operations due to their high density, clay inhibition and low solid content. However, there is a lack of thickeners to promote gel strength, which improves the fluid’s capacity to carry and suspend cuttings. To solve this problem, the branched polymer (hereafter abbreviated as PAD-B) was prepared by the copolymerization of N,N-dimethylacrylamide (DMAM) and 2-acrylamide-2-methylpropane sulfonic acid (AMPS), using polyethylenimine as a branching agent and cerium ammonium nitrate as the initiator. Compared with linear polymer (PAD-L), PAD-B has better shear strength at the same low viscosity. The experimental results indicated that the increase in shear strength of PAD-B is due to the interactions between branched PAD-B molecules, which lead to the formation of a network structure. The effect of calcium chloride (CaCl_2_) on the rheological performance of PAD-B was investigated at 25 °C and 50 °C. Compared with PAD-L, PAD-B shows better thermal stability and calcium resistance. Its high gel strength provides technical support for addressing issues such as low yield point, gel strength and difficulty in controlling the rheological parameters of calcium halide-based fluids during the drilling and completion of complex wells.

## 1. Introduction

In drilling engineering, drilling fluid serves several functions, such as cooling the bit, balancing formation pressure and obtaining geological information. However, the most important function is to carry cuttings back to the surface and keep the hole clean [1,2,3]. In addition to the influence of drilling equipment, the rheology of drilling fluid significantly affects cuttings carrying. Among the rheological parameters of drilling fluid, yield point (YP) is the most important parameter for characterizing its cuttings-carrying capacity [4]. In brief, YP represents the gravitational attraction between colloidal particles in circulating drilling fluid, that is, it reflects the strength of the network structure within the drilling fluid under dynamic conditions. The higher the YP value, the greater the rock-carrying capacity of drilling fluid, and the more easily cuttings at the bottom of the well are pushed to the surface [5,6,7].

In drilling operations, to improve the YP value of drilling fluid, engineers typically add bentonite, which is the most cost-effective approach [8]. However, if the bentonite content in drilling fluid is too high, it can lead to negative effects such as mud-coated bit, density fluctuations, an increase in solid phase content, and higher fluid consumption [9,10]. According to the literature, the transport capacity of cuttings is typically improved by enhancing the rheological properties of drilling fluids through the addition of polymers, including xanthan gum, carboxymethyl cellulose, polyanionic cellulose, and other natural products, as well as synthetic polymers such as polyacrylamide and its derivatives [11,12,13,14,15]. However, excessive plastic viscosity (PV) in drilling fluid negatively impacts the rate of penetration, degassing efficiency, surging pressure, circulation pressure consumption, mud cake quality, and so on [16,17]. Therefore, one of the important roles of polymeric additives is to improve the cuttings-carrying capacity of drilling fluid without significantly affecting its viscosity. Furthermore, calcium halide-based fluids are often used in drilling and completion operations due to their high density, clay inhibition, and low solid content. However, there is a lack of thickeners to promote gel strength and improve the cuttings carrying and suspending capacity of these fluids.

Highly branched polymers (HBPs) are highly branched macromolecules with a three-dimensional architecture; also, the molecular chain of HBP is hard to tangle and easy to graft [18,19]. Due to its small fluid mechanical volume, determined by its spherical molecular structure, branched polymers have advantages in temperature and salt resistance as well as compactness. Moreover, branched polymers have a large number of modifiable terminal groups, which give them valuable properties such as good solubility, low viscosity and high reactivity [20,21]. An increasing number of studies have focused on the potential value of branched polymers in the oil and gas industry [22]. The amide polymers enhance the YP value of drilling fluid by forming hydrogen bonds between amide groups and other functional groups in the molecule. In addition, the interaction between hydroxyl and carbonyl groups in the molecules and other groups further strengthens the structure of the network, resulting in a drilling fluid system that shows higher YP and suspension performance [23,24,25,26]. For example, Jiang et al. synthesized a ternary copolymer ZJA, the supermolecular three-dimensional network formed among the ternary copolymers can be observed through TEM, ZJA also has a good salt resistance at 150 °C and can be applied in the saturated salt-water drilling fluid [27].

**Scheme 1:**
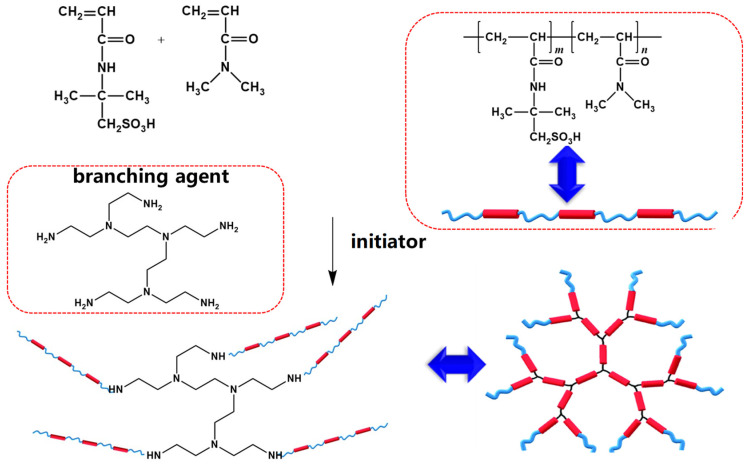
Synthesis route of branched polymer PAD.

In this study, a thickening polymer for calcium chloride-based drilling fluid, with a low viscosity and high gel strength additive (PAD-B), was synthesized by copolymerization of N, N-dimethylacrylamide (DMAM) and 2-acrylamide-2-methylpropane sulfonic acid (AMPS) in aqueous solution, using polyethylenimine as a branching agent and ammonium cerium nitrate as an initiator (Scheme 1). The molecular structure and rheological properties of PAD-B were determined, and their effects on rheological properties were compared with those of linear polymer PAD-L. By introducing strong hydration groups (such as sulfonic acid groups) to maintain hydration dispersion in a high-salt environment, PAD-B can be self-assembled in a salt environment so that the entire solution forms a three-dimensional spatial network structure with strong shear strength due to the interaction between the functional groups of PAD-B, allowing PAD-B to effectively improve the YP value of water-based drilling fluids. It solves the defect that the increase in plastic viscosity of water-based viscosifiers and shear strength improver is generally higher than that of shear strength. The PAD-B systems with CaCl_2_ were investigated for their rheological performance at 25 °C and 50 °C. After the addition of Ca^2+^ ions, the original state of polymer molecules in the solution is disrupted, and the interaction between the molecular chains is weakened, thus reducing the strength of the network structure within the drilling fluid. The change in the viscoelasticity of PAD-B is smaller than that of PAD-L after the addition of CaCl_2_ and heating, indicating that PAD-B shows better thermal stability and salt resistance compared to PAD-L. This work developed a branched polymer with low viscosity and high gel strength that can be used as a water-based drilling fluid resistant to high temperatures and high salinity. This research provides technical insights for the development of rheology modifiers.

## 2. Results and Discussion

### 2.1. Structural Analysis of PAD-B

**Figure 1:**
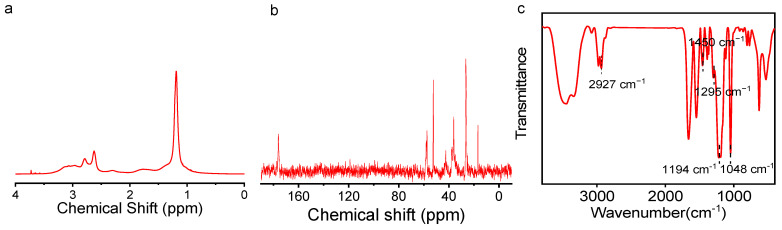
(**a**) ^1^H NMR spectrum, (**b**) ^13^C NMR spectrum and (**c**) FTIR spectrum of PAD-B.

The structure of PAD-B was confirmed by NMR and FT-IR analysis, as shown in Figure 1. In Figure 1a, the chemical shift around 2.7–3.4 ppm is attributed to the methylene protons (-CH_2_- of -CH_2_SO_3_^−^) from the AMPS unit, while the methyl protons (-CH_3_ of -C-CH_3_) from the AMPS unit contribute to the signal around 1.2 ppm. The methyl proton (-CH_3_ of CH_3_-N-) of the DMAM unit also appears around 2.5–2.9 ppm. The ^13^C NMR of PAD-B is shown in Figure 1b. The chemical shifts at 26.46 and 42.43 ppm represent C in the monomers (DMAM, AMPS) in PAD-B. The chemical shifts at 52.43 and 57.19 ppm are the C on –NH-CO- in AMPS. The chemical peak at 176.19 ppm comes from C in the C=O in monomers (DMAM, AMPS). In Figure 1c, the characteristic peaks around 3400–3500 cm^−1^ (O-H), 1650–1750 cm^−1^ (C=O), 1194 cm^−1^ (S=O), 1048 cm^−1^ (S=O), and 600–700 cm^−1^ (S-O) are identified as the absorption band of sulfonic groups. The peaks at around 2927 cm^−1^ and 1450 cm^−1^ are for the C-H bond and 1295 cm^−1^ for the C-N bond, which could be due to the presence of DMAM unit in the polymer. The values of the weight average molecular weight for PAD-B and PAD-L were determined by LLS measurement, and the results are 6.01 × 10^6^ g/mol and 2.86 × 10^6^ g/mol, respectively.

### 2.2. Rheological Properties of PAD-B/PAD-L

The preparation of drilling fluid needs to have good thixotropy; that is, when stirring, the suspension appears as a sol with good fluidity, and after stopping stirring, it will automatically arrange into a gel with a network structure. Similarly, no precipitation and water separation occurred during the preparation of the drilling fluid. Based on these conditions, to prepare high-performance drilling fluid, we selected 45 mg·mL^−1^ PAD-B as the typical sample.

**Figure 2:**
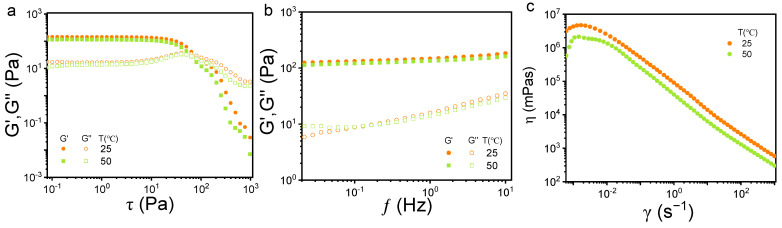
Rheological results for PAD-B system at different temperatures. (**a**) G′ and G″ as a function of the applied stress at a constant frequency (1.0 Hz). (**b**) Variations of G′ and G″ as a function of frequency. (**c**) Variation of shear viscosity as a function of shear rate.

Figure 2 presents the results of the rheological measurements of the PAD-B system to reveal its viscoelastic properties. Initially, the relationship between moduli and strain can be obtained from the dynamic strain sweep experiment. Distinct linear viscoelastic regions can be observed for the PAD-B system at different temperatures. It can be observed that the storage modulus G′ (~131.82 Pa) is bigger than the loss modulus G″ (~14.81 Pa) in the linear viscoelastic region at 25 °C, indicating that the elasticity is more outstanding than viscosity, with stress (σ) increasing to the critical stress value (σc) of 83.39 Pa, and G″ is higher than G′. The stress value marks the point where the viscoelastic structure begins to break down and enters the nonlinear flow region. The greater the limit of the linear viscoelastic region, the greater the capacity to resist stress up to a specific threshold before undergoing irreversible deformation. It may be the network structure forms via intermolecular interactions of polymer chains of PAD-B, and the network is destroyed when the temperature is increased [28,29]; the corresponding linear viscoelastic region is smaller (σc = 64.42 Pa). According to the viscoelastic region we obtained for each sample, we performed the oscillatory shear sweep frequency measurement at a constant level of stress (0.5 Pa). According to the oscillatory shear frequency sweep measurements, the PAD-B system at 25 °C showed that storage modulus G′ (~136.91 Pa) and loss modulus G″ (~10.22 Pa) remain constant and G′ > G″, which confirms the above gel-like rheological behavior [30]. As far as the dynamic strain sweep and the oscillatory shear sweep frequency measurements are concerned, they follow a similar rheological pattern: with the increase in temperature, the decrease in viscoelasticity is small, which indicates that the branched polymer PAD-B has good temperature resistance.

The shear rate dependence of the apparent viscosity (η) for the PAD-B system at different temperatures (Figure 2c) was exhibited. The PAD-B system exhibited a shear-thinning effect at higher shear rates. The first few points at low shear rates are mainly due to elastic startup effects due to a contrast between the sample preparation (high shear) conditions immediately prior to placing the sample in the rheometer and those for the initial low shear measurements [31]. As the shear rate increases, the typical behavior of plastic fluids is observed [30]. When other conditions are held constant, the viscosity of the PAD-B system decreases with the increasing temperature. This is because warming enhances the activity of PAD-B molecules and water molecules, weakens the hydrogen bonds, and reduces the internal friction between water molecules, PAD-B molecules, and between water and PAD-B molecules, resulting in a decrease in the viscosity of the entire system. However, the apparent viscosity decreases less significantly when the temperature rises to 50 °C, which further indicates that the PAD-B molecules exhibit advantages in temperature resistance.

**Figure 3:**
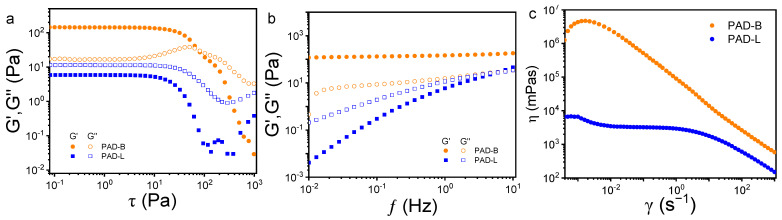
Rheological results for PAD-L system at 25 °C. (**a**) G′ and G″ as a function of the applied stress at a constant frequency (1.0 Hz). (**b**) Variations of G′ and G″ as a function of frequency. (**c**) Variation of shear viscosity as a function of shear rate.

The rheology of the PAD-L system at the same concentration is shown in Figure 3. Moreover, the loss modulus G″ was greater than the storage modulus G′ in the linear viscoelastic region, indicating that the viscosity was more prominent than elasticity, which suggested that it does not exhibit gel-like properties. As the stress increases, G′ decreases much faster than G″, indicating that the elastic property can be more easily altered by applied stress. The oscillatory shear frequency sweep measurements showed that dynamic moduli (G′ and G″) increase with increasing frequency, with different slopes, but always remain G″ > G′.

The frequency dependence of the apparent viscosity for the PAD-L system differed from that of the PAD-B system (Figure 3c). From steady shear measurements, a plateau was detected for the apparent viscosity of the PAD-L system at low shear rates. In the region of high shear stresses, a decrease in viscosity results from the disentanglement of macromolecular chains—these rheological features are also characteristic of traditional linear polymers [32]. Similarly, when other conditions are held constant, the viscosity of the PAD-L system decreases with increasing temperature. However, the shear thinning behavior of the PAD-L system is limited, which is not conducive to improving the drilling rate or to suspending and carrying drilling cuttings.

### 2.3. The Effect of CaCl_2_ on the Rheological Measurements of PAD-B

**Figure 4:**
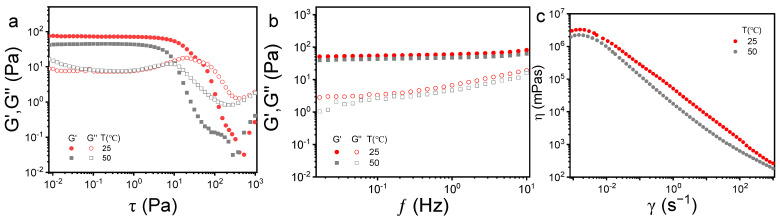
Rheological results for PAD-B/CaCl_2_ system at different temperatures. (**a**) G′ and G″ as a function of the applied stress at a constant frequency (1.0 Hz). (**b**) Variations of G′ and G″ as a function of frequency. (**c**) Variation of shear viscosity as a function of shear rate.

As shown in Figure 4, after adding CaCl_2_, PAD-B still exhibited a higher storage modulus than loss modulus within the linear viscoelastic region, implying that the elastic component constitutes a larger proportion of the solution compared to its viscous component. At constant stress (0.5 Pa) as a function of frequency, we carried out oscillatory measurements and found that G′ is still higher than G″. However, both G′ and G″ of PAD-B decrease with the addition of CaCl_2_. At 25 °C, G′ (~74.21 Pa) and G″ (~7.39 Pa) of PAD-B/CaCl_2_ showed 50% of its original apparent viscosity. The effect of CaCl_2_ on G′ and G″ may be ascribed to the interaction between the introduction of Ca^2^⁺ ions and the polymer, which changes the interaction between polymer molecular chains of PAD-B. As a result, the water molecules surrounding the molecular chain form a highly ordered solvation layer connected by hydrogen bonds, causing the polymer to exhibit a stretched structure [33]. After CaCl_2_ is added, the Ca^2+^ cation charge density is relatively high, the electric charges on the polar groups of PAD-B are shielded, the original state of polymer molecules in water is broken, the presence of Ca^2^⁺ can hinder the formation of hydrogen bonds within the molecule, which subsequently influences the polymer’s behavior, resulting in the molecular curl, the viscoelasticity is reduced, so G′ and G″ of the PAD-B/CaCl_2_ system is lower than those of PAD-B system. After the temperature rose, the dynamic strain sweep and the oscillatory shear sweep frequency measurements showed that temperature has a small effect on the viscoelasticity of the PAD-B/CaCl_2_ system, indicating that the PAD-B system still has a good temperature resistance after the introduction of CaCl_2_.

At higher shear rates, the viscosity of the PAD-B/CaCl_2_ system decreased with an increasing shear rate, which indicates a shear-thinning effect consistent with the typical behavior of plastic fluids. As the temperature rises, the distance between the molecules increases, decreasing the attraction between them, which reduces internal friction decreases and leads to a decrease in viscosity for PAD-B/CaCl_2_ system. However, the viscosities of PAD-B system are always higher than that of PAD-B/CaCl_2_ system at the same temperature, implying that the introduction of CaCl_2_ disrupts the interaction between PAD-B molecules.

### 2.4. The Effect of CaCl_2_ on YP and PV of PAD-B

**Figure 5:**
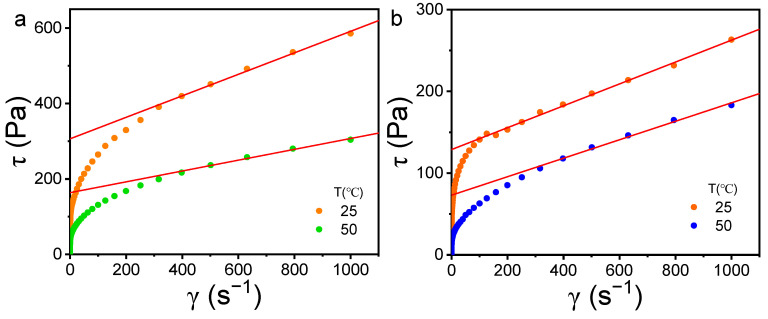
The shear stress as a function of shear rate at different temperatures: (**a**) 45 mg·mL^−1^ PAD-B/CaCl_2_; (**b**) 45 mg·mL^−1^ PAD-B/CaCl_2_.

Figure 5 shows the shear stress as a function of shear rate of PAD-B without and with CaCl_2_ at different temperatures. The rheological curve of plastic fluid is a curve at the stage of low shear rate, and the viscosity decreases with the increase in shear rate. When the shear rate increases to a certain value, the rheological curve presents a straight line, and the fluid viscosity no longer changes with the change in shear rate, the slope of this line segment is the plastic viscosity (PV), and the shear stress value at the intersection of its inverse extension line and shear stress axis is called YP. These parameters are usually used in the Bingham model to present its constitutive relationship of drilling fluid and equations of these parameters are given in the articles [4].

$$\tau =\tau_{0}+\mu_{p}\gamma$$

where τ = shear stress of the drilling fluid (Pa); $\mu_{p}$ = PV (mPa·s); $\tau_{0}$ = YP (Pa). In general, YP is the resistance to moving the fluid initially, which is a crucial rheological property that influences the parameters of drilling fluid. PV is the resistance when the fluid moves freely, this resistance is caused by friction between suspended solid particles, between solid particles and continuous phase, and within continuous phase in the drilling mud.

As shown in Table 1, at the same temperature, the YP and PV values of PAD-B decrease after the addition of CaCl_2_. The introduction of salt ions leads to a change in the molecular chain structure within the polymer solution, the screening effect that occurs on the negative charges of the polar groups in the polymer due to the presence of Ca^2^⁺ ions. This screening reduces the electrostatic repulsion between the polymer chains, significantly decreasing the PV value of PAD-B. The YP values of drilling fluid decrease with the increase in temperature, mainly because the hydrogen bonding between polymer molecular chains of PAD-B in the system weakens with the increase in temperature, and the structural strength of the system becomes weaker. In consequence, the YP values of systems decrease in the presence of CaCl_2_ when the temperature is increased, but after the introduction of CaCl_2_, the attenuation of YP with increasing temperature decreased.

### 2.5. Comparative Evaluation Experiment

#### 2.5.1. Comparison of YP and PV with PAD-L

**Figure 6:**
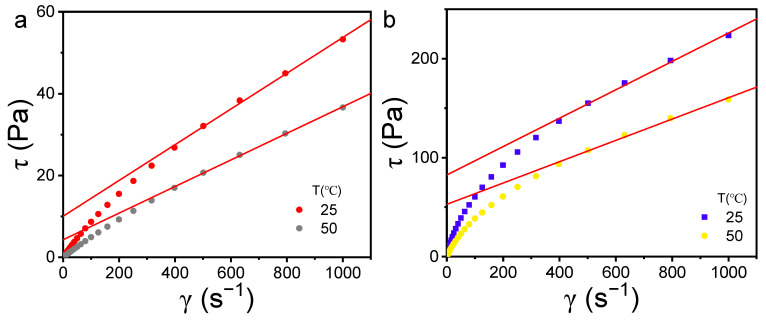
The shear stress as a function of shear rate at different temperatures: (**a**) 45 mg·mL^−1^ PAD-L + CaCl_2_; (**b**) 112.5 mg·mL^−1^ PAD-L + CaCl_2_.

Figure 6 shows the relationship between shear stress and shear rate for PAD-L/CaCl_2_ at different temperatures and concentrations. As shown in Table 1, when the amounts of PAD-L and PAD-B were the same (45 mg·mL^−1^), the YP and PV values of PAD-L/CaCl_2_ were lower than those of PAD-B/CaCl_2_ at different temperatures. At 25 °C, the YP value of PAD-B/CaCl_2_ (128.40 Pa) is 12.8 times greater than that of PAD-L/CaCl_2_, while the PV value (134.45 mPa·s) is also 3.07 times higher. When the temperature reaches 50 °C, the YP value of PAD-B/CaCl_2_ (73.04 Pa) is 21.4 times greater than that of PAD-L/CaCl_2_, whereas the PV value (112.96 mPa·s) is 3.4 times greater. These results demonstrate that the PV and YP values of PAD-B/CaCl_2_ are higher than those of PAD-L/CaCl_2_, with the increase in YP being more significant. This indicates that PAD-B/CaCl_2_ has a smaller impact on plastic viscosity than PAD-L/CaCl_2_ at different temperatures. We speculate that the superior capacity of PAD-B/CaCl_2_ is due to the shear resistance brought by the network structure forms via intermolecular interactions of polymer chains of PAD-B. We increase the amount of PAD-L added to the CaCl_2_ solution, resulting in an increase in both the YP and PV values. When the concentration of PAD-L/CaCl_2_ is raised to 2.5 times the original concentration at 25 °C, the PV value (134.45 mPa·s) becomes comparable to that of PAD-B/CaCl_2_ (143.42 mPa·s). However, the YP value (82.56 Pa) remains lower than that of PAD-B/CaCl_2_ (128.40 Pa). This shows that even if the concentration of PAD-L is increased, the shear resistance is still weaker than that of PAD-B at low concentrations. A similar trend is observed when the temperature rises to 50 °C, which demonstrates that the addition of PAD-B enhances the shear strength of drilling fluid.

#### 2.5.2. Comparison of Rheological Measurements with PAD-L

**Figure 7:**
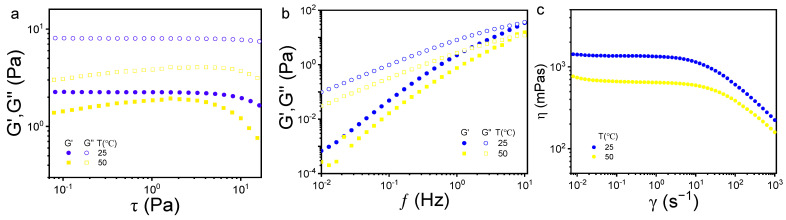
Rheological results for 112.5 mg·mL^−1^ PAD-L+CaCl_2_ system at different temperatures. (**a**) G′ and G″ as a function of the applied stress at a constant frequency (1.0 Hz). (**b**) Variations of G′ and G″ as a function of frequency. (**c**) Variation of shear viscosity as a function of shear rate.

For the 112.5 mg·mL^−1^ PAD-L/CaCl_2_ system, the dynamic strain sweep experiment conducted at different temperatures indicated that G′ and G″ are nearly independent of stress within a certain stress range (Figure 7a). Moreover, G″ is greater than G′ in the linear viscoelastic region, indicating that the viscosity is more pronounced than elasticity. At different temperatures, the frequency dependence of G′ and G″ for 112.5 mg·mL^−1^ PAD-L/CaCl_2_ system showed typical liquid-like behavior, with G″ > G′. In comparison to the 45 mg·mL^−1^ PAD-B/CaCl_2_ system, the reduced viscoelasticity in PAD-L is due to a lack of significant chain entanglement and a lower 3D network density compared to the branched PAD-B system, which leads to the weak interaction between the molecular chains of the PAD-L/CaCl_2_ system and the low stiffness of the three-dimensional network structure in the drilling fluid. Additionally, the decrease in G′ and G″ in the PAD-B/CaCl_2_ system are often smaller than those in the PAD-L/CaCl_2_ system when the temperature increases. Therefore, it can be concluded that the PAD-B/CaCl_2_ system has a better ability to withstand temperature changes compared to the PAD-L/CaCl_2_ system. From stable shear measurements, the apparent viscosity of the 112.5 mg·mL^−1^ PAD-L/CaCl_2_ system is detected as a plateau at low shear rates, with viscosity decreasing in regions of high shear stress. Similarly, the shear thinning behavior of the 112.5 mg·mL^−1^ PAD-L/CaCl_2_ system is poor, which makes it inapplicable to drilling fluid systems.

### 2.6. Morphology Characterization

**Figure 8:**
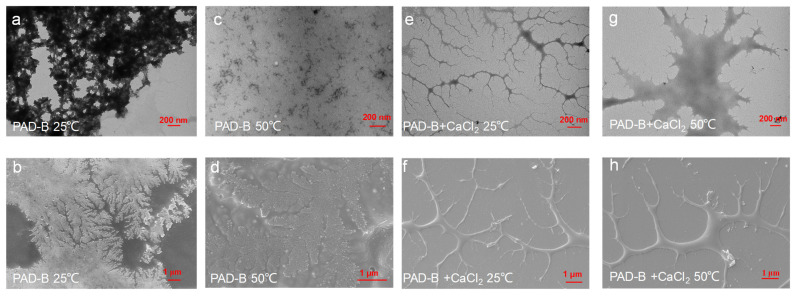
Microstructure of the PAD-B system with or without CaCl_2_ at different temperatures: (**a**,**c**,**e**,**g**) TEM, (**b**,**d**,**f**,**h**) SEM.

To study the action mechanism of PAD-B in drilling fluid, transmission electron microscopy (TEM) and scanning electron microscopy (SEM) images were used to clearly characterize the micro-morphology of PAD-B (Figure 8). In an aqueous solution of PAD-B, a micro-crosslinking network is formed. Due to the hydrogen bonding, the water molecules surrounding the molecular chains form a highly ordered solvation layer. This solvation layer benefits from the intermolecular hydrophobic association of PAD-B chains and the combined action of hydrogen bonds between pendent groups in the polymer chain, resulting in the formation of this network structure and aggregation, which exhibit good shear strength. With the increase in temperature, the hydrophilic regions lose hydrogen bonds, and the increased mobility of water molecules at higher temperatures weakens solvation, and the aggregation of molecules is reduced.

It can be seen from Figure 8 that after the introduction of CaCl_2_, the continuous network of PAD-B is destroyed at different temperatures, but a large number of aggregates of varying sizes form and connect with each other, creating a small network structure. This indicates that the intermolecular association of hydrophobic groups and pendent groups has a positive effect on the internal structural strength.

## 3. Materials and Methods

### 3.1. Materials

N,N-dimethylacrylamide (DMAM, 99%), 2-acrylamide-2-methylpropane sulfonic acid (AMPS, 98%), polyethylenimide with a molecular weight of 1800 g/mol (PEI, 99%), ammonium cerium nitrate (CAN, 98%), 2,2′-azobis(2-methylpropionamidine) dihydrochloride (AIBA, 97%) and calcium chloride (CaCl_2_, 96%) were purchased from Shanghai Aladdin Biochemical Technology Co. Ltd. and used as received.

### 3.2. Preparation of Polymers

The preparation processes for PAD-B are as follows [34,35]. First, 16 g of AMPS, 4 g of DMAM, and 0.2 g of PEI were added to 80 g of deionized water, followed by stirring until all chemicals were fully dissolved. Next, the solution was added into a three-neck flask and stirred under a N_2_ atmosphere for 30 min, and its temperature was adjusted to 45 °C. Then 0.3 g of CAN was introduced into the solution, and the polymerization was maintained for 6 h. The product was cut into small pieces, washed by ethanol/water (*v*/*v*, 7/3) twice and CH_3_CH_2_OH twice, sieved, and dried in vacuo at 60 °C for 24 h. PAD-L used as contrast sample was also synthesized as follows. First, 16 g of AMPS and 4 g of DMAM were added to 80 g of deionized water, followed by stirring until all chemicals were fully dissolved. Next, the solution was added into a three-neck flask and stirred under a N_2_ atmosphere for 30 min, and its temperature was adjusted to 45 °C. Then 0.1 g of AIBA was introduced into the solution, and the polymerization was maintained for 6 h. The product was cut into small pieces, washed by ethanol/water (*v*/*v*, 7/3) twice and CH_3_CH_2_OH twice, sieved and dried in vacuo at 60 °C for 24 h.

### 3.3. Preparation of Sample Solutions

Add a series of different amounts of PAD-B/PAD-L (45 mg·mL^−1^) to the 300 mg·mL^−1^ CaCl_2_ solution and pure deionized water, then stirred until completely dissolved, and the samples were then stored overnight in a thermostat at 25 °C to equilibrate.

### 3.4. Instruments

The ^1^H NMR spectra were measured on a Bruker AVANCE III HD 400 MHz spectrometer (USA) at room temperature, and D_2_O was used as the solvent for the sample. Fourier-transform infrared (FT-IR) spectra were measured on a Tensor II spectrophotometer (Bruker, Germany) while samples were carried with KBr powder. Laser light scattering (LLS) measurements were performed using the multi-detector light scattering unit (DAWN HELEOS, Wyatt Technology Corporation, US). The polymer was dissolved in 0.1 mol/L NaCl. The sample solution was filtered through Millipore 0.8 μm hydrophilic membranes before use. The steady and dynamic shear rheological properties were determined using a Haake MARS 60 rheometer (Germany) with a sensor stainless steel cylinder Cup CCB25 (27 mm diameter) and a sensor coaxial rotor CC25/Ti, respectively. In oscillatory measurements, an amplitude sweep at a fixed frequency of 1 Hz was performed prior to the following frequency sweep to ensure that the selected stress was in the linear viscoelastic region. In experiments aiming to see a temperature influence, the temperature was typically secured with the help of a cyclic water bath. After each temperature rise, the sample was allowed to equilibrate for an additional 5 min before measurements. The microstructure images were obtained on JCR-100CX II TEM (transmission electron microscope, JEOL, Japan) and JSM-7800F FE-SEM (field emission scanning electron microscope, JEOL, Japan). A copper mesh was inserted into the gel/solution to obtain a sample and, after drying under an IR lamp for 45 min, TEM images were observed under a JCR-100CXII(JEOL) microscope. The gel/solution was placed on a silica wafer, dried for 45 min under an IR lamp, and observed by field-emission SEM.

## 4. Conclusions

In this study, a novel branched copolymer poly(*N*,*N*-dimethylacrylamide, and 2-acryloylamino-2-methyl-1-propanesulfonic acid) was synthesized and named PAD-B. PAD-B had a low viscosity and high gel strength. In solution, PAD-B showed good dispersibility, and hydrophilic chain segments interacted well with the aqueous medium, forming an extended structure according to environmental conditions; thus, the whole solution forms a three-dimensional spatial network structure through the interaction between functional groups, which can significantly increase the shearing force. The PAD-B systems with CaCl_2_ were investigated via rheological performance at 25 °C and 50 °C. The addition of Ca^2+^ ions leads to changes in the structure of the molecular chains of PAD-B. Adding CaCl_2_ and heating up can effectively reduce the PV value. Owing to the network structure forms via intermolecular interactions of polymer chains of PAD-B, the capability of the PAD-B system to endure higher temperatures is better than that of the PAD-L system. The synthetic process is simple and easy, and has good application prospects. It is suitable for use as a rheology modifier for solid-free water-based drilling fluids.

## Figures and Tables

**Table 1:** Performance test of different drilling fluids at different temperatures.

Samples	YP (τ_0_, Pa)	PV (μ_p_, mPa·s)
PAD-B at 25 °C	306.47	285
PAD-B at 50 °C	163.62	143.72
PAD-B/CaCl_2_ at 25 °C	128.40	134.45
PAD-B/CaCl_2_ at 50 °C	73.04	112.96
PAD-L(+1×/CaCl_2_ at 25 °C	10.04	43.70
PAD-L(+1×)/CaCl_2_ at 50 °C	3.41	33.66
PAD-L(+2.5×)/CaCl_2_ at 25 °C	82.56	143.42
PAD-L(+2.5×)/CaCl_2_ at 50 °C	48.94	112.64

## Data Availability

Data are contained within this article.
